# Supporting students with complex needs living in rural and regional New South Wales: is wraparound the answer?

**DOI:** 10.1007/s13384-022-00570-z

**Published:** 2022-09-26

**Authors:** Therese M. Cumming, Iva Strnadová, Lisa Gilanyi, Hee Min Lee

**Affiliations:** 1 UNSW Sydney, Sydney, NSW Australia; 2 UNSW Disability Innovation Institute, Sydney, NSW Australia

**Keywords:** Wraparound models, Complex support needs, Rural and remote schooling, Multisystemic system of support

## Abstract

Historically, students attending school in rural and regional New South Wales have experienced poorer outcomes than their peers attending metropolitan schools. The lack of coordinated support services for students with complex support needs compounds this issue. Wraparound models of support have been successful in improving outcomes for students with complex support needs, and the New South Wales government has prioritised the establishment of strong relationships between schools and communities to overcome the limitations of geographic isolation. The aim of the current study was to explore wraparound support for students with complex support needs attending schools in rural and regional New South Wales. A qualitative research approach was employed, and semi-structured interviews were conducted with key stakeholders to gain an in-depth understanding of current successes, barriers, and needs. The findings indicated that wraparound was most effective in rural and remote schools when school staff implemented bespoke approaches to wraparound, such as restorative practices. Resourcing was a barrier found to be central to all schools. Recommendations are provided to enhance the capacity of rural and regional NSW schools to provide wraparound support for students with complex support needs.

## Introduction

Students attending school in rural and regional New South Wales have experienced poorer outcomes than their peers attending metropolitan schools (Halsey, 2018). A lack of support services for a growing population of students with complex support needs (those with disabilities, mental health issues, and social disadvantage) compounds this issue (Dowse et al., 2014). When left unmet, these needs increase students’ risk of poor outcomes, including educational disengagement, precarious housing, substance misuse, and involvement with the juvenile justice system (Cumming et al., 2019). Although many students with complex support needs receive a variety of services both in and outside of school, a lack of central coordination of these services often leads to gaps in and overlap of supports, resulting in poor outcomes for the students (Yu et al., 2020). However, research demonstrates that the provision of wraparound services has the potential to improve outcomes for students with complex support needs (Yu et al., 2020).

Wraparound was developed in the United States in the fields of mental health and child welfare. It was developed as a team-based, collaborative process for developing and implementing individualised care plans for young people with, and at-risk of, emotional and behavioural disabilities, and their families (Walker & Bruns, 2006). Bruns and Walker (2008) developed core principles to underpin wraparound support, the most significant being that wraparound is not a single service, but instead a collaborative process through which specific school and community-based interventions are designed, implemented, and coordinated. A team comprising the student, family members, natural supports (e.g. extended family, friends, mentors), and school and community professionals is assembled. The team then collaborates to develop a support plan that is student- and family-centred, as it addresses their needs and priorities in a way that is acceptable to them.

In Australia, although collaborative systems have been emerging, there is a lack of focus on the education sector in this area, because wraparound has evolved from the earlier mental-health-related ‘system of care’ models (Winters & Metz, 2009). Education providers have tended to be peripheral to the primary aims of mental health-led teams assembled in support of students with complex support needs. However, some scholars have also explored the distinctive and structured approach that wraparound provides to school and support service collaboration. Wyles (2007) developed a wraparound program called *Turnaround* in the Australian Capital Territory. The program was a new model of providing case coordination to young people with complex issues. It highlighted the case coordination bringing together a range of service providers encompassing all the services required to assist the young person.

Wraparound is outcomes-based, with long-term goals centred around home-like placements and improved quality of life, demonstrated through improved functioning in school, the community, and vocationally (Hill, 2020). A unique team is chosen based on the needs of the young person and may include professionals from education, mental health, juvenile justice, employment, community services, government, and medicine. A designated team facilitator (often a school social worker, counsellor, or psychologist) guides the team through the wraparound process for as long as it takes to meet the needs of the young person and their family (Yu et al., 2020). The principles of wraparound are well aligned with Bronfenbrenner’s ecological systems theory (Bronfenbrenner, 1994).

## Theoretical framework

Today’s schools contain many students with challenging behaviours and complex support needs, and for some of these students these behaviours are ongoing, despite the implementation of evidence-based interventions (Farmer, 2013). The broadening of the application of wraparound beyond the medical model has necessitated an ecological perspective (e.g. Farmer et al., 2016; Savina et al., 2014), specifically Bronfenbrenner’s ecological systems theory (Bronfenbrenner, 1994).

The principles of wraparound are consistent with ecological systems theory, as an individual’s behaviour develops in the context of many reciprocal interactions over time (Bronfenbrenner, 1994). These relationships contribute to an individual’s behaviours in a developmental process. Therefore, any kind of support for behaviour change needs to happen within and through interactions within those settings. Bronfenbrenner’s (1994) theory supports the individualised care for youth with complex needs, as it stresses the unique relationships between the child and the various environmental systems (family, community, school). This theory can be used to guide intervention, as an understanding of each young person’s social, cultural, and interpersonal systems environment and supports must be tailored to each young person’s unique set of relationships.

**Fig. 1:**
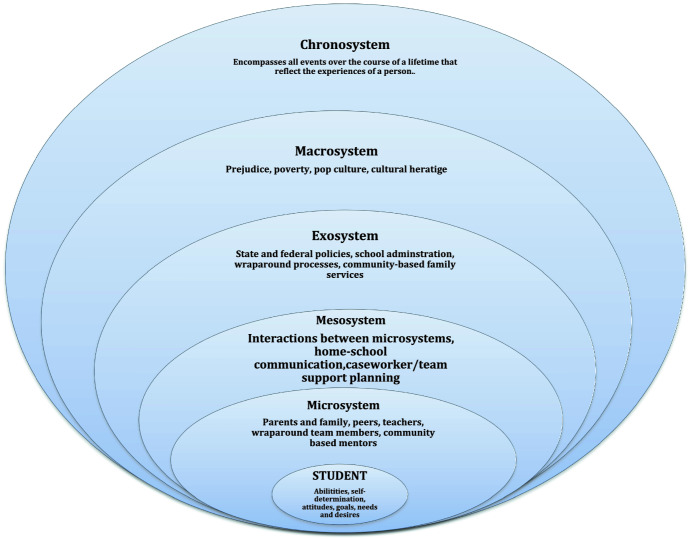
Bronfenbrenner's ecological systems theory

Through the lens of ecological systems theory, the wraparound for an individual young person can be viewed as reaching inwards to the microsystems of the school and outwards beyond the school (see Fig. 1 for an illustration). The support required for the student may include collaboration with professionals working in systems beyond the school, ideally in the form of a physical “inreach” into the school system, although outreach to agencies in the local community and beyond may be required (McIntosh et al., 2010; Messina et al., 2015). This would be representative of the student’s mesosystem. State and Federal policies, particularly around funding, affect the student on an exosystem level, and may enable or hinder the student being able to access necessary supports (McIntosh et al., 2010). The macrosystem, consisting of the views of society and cultural norms, would also affect what services the student and their family might access or how the student functions in relation to others’ perceptions of them (Bronfenbrenner, 1994). Lastly, Bronfenbrenner (1994) explained that the chronosystem reflects all systems and the student’s experiences over a lifespan and how they shape who they are.

## New South Wales rural and remote education

The New South Wales (NSW) Government (2013) recognises that NSW, like Australia in general, has a larger “remoteness gap” than the average of other OECD nations. This gap is also larger in NSW than almost any other Australian state or territory, leading to disadvantage for many students in rural and remote NSW. This disadvantage begins in early childhood and continues through to school outcomes such as achievement tests, attendance, and high school completion. *Rural and Remote Education: A Blueprint for Action* (NSW Government, 2013) was a reform agenda comprising evidence-based initiatives designed to improve the outcomes of students in rural and remote schools by exposing them to the same opportunities and teaching practices as their metropolitan peers.

For the purposes of this study, the terms ‘rural’ and ‘remote’ are used generally to refer to non-metropolitan areas. However, the four towns included in the current project can be further classified using the Australian Bureau of Statistics Australian Statistical Geographical Standard (ASGS), which divides Australia into five classes of remoteness based on relative access to services (Australian Bureau of Statistics, 2018): metropolitan, inner regional, outer regional, remote or very remote. Two towns were classified as inner regional and two were outer regional.

The aim of the current study was two-fold. First, we wanted to establish the extent to which wraparound was already being implemented in rural and remote schools. The second part was to identify the enablers, barriers, and additional needs for effective wraparound support for students with complex needs. Interviews were conducted with key stakeholders in six schools, located in four towns in rural or remote areas of NSW to discover the answers to the following research questions:

1. What are the perceived wraparound support needs of students with complex needs in rural and remote schools as reported by stakeholders?
2. What are the enablers of effective wraparound support in rural and remote schools as reported by stakeholders?
3. What are the barriers to effective wraparound support in rural and remote schools as reported by stakeholders?

## Method

### Research design

The authors applied for and received ethics approval from the human research ethics committees at their university and the state education authority. The authors employed a qualitative research design to gain an in-depth understanding of the experiences and perceptions of the stakeholders involved in wraparound support in regional NSW schools (Bengtsson, 2016). Principals, deputy principals, head teachers, specialist teachers, classroom teachers, and school counsellors were interviewed separately and in small groups, using a semi-structured interview protocol that was developed based on a critical review of literature focussed on wraparound models and their implementation (Cumming et al., 2022). The interviews were audio-recorded with the participants’ permission, transcribed verbatim, and analysed using a qualitative thematic analysis approach (Braun & Clarke, 2006).

### Participants and setting

A total of 18 interviews were conducted (2 with small groups and 16 individual), with 24 interviewees. The participants ranged in age from 27 to 63, and the majority were female (11). See Table 1 for detailed participant demographic information. Pseudonyms were assigned to each participant to preserve anonymity.

**Table 1:** Participant demographic information

Participant	Age	Gender	Position	Time working with youth with complex needs (years)	Highest level of education	Participation in professional learning?	Professional learning in the area of wraparound
DCS1	39	Female	Head teacher—wellbeing	7	Bachelor’s Degree	Yes	No
DCS2	30	Female	School learning support officer	5	Bachelor’s Degree	Yes	Yes
DCS3	45	Female	Head Teacher Special Ed	13	Graduate Diploma	Yes	Yes
DCS4	48	Female	Deputy Principal	25	Master’s Degree	Yes	No
DCS5	29	Female	School psychologist	3	Master’s Degree	Yes	No
DSN1	34	Female	Classroom teacher	4	Bachelor’s Degree	Yes	No
DSN2	46	Female	Head teacher Special Ed	20	Bachelor’s Degree	Yes	Yes
W1	35	Female	Head Teacher of Support	10	Master’s Degree	Yes	No
W2A	54	Female	Deputy Principal	31	Graduate Diploma	Yes	No
W3A	63	Male	Principal	33	Bachelor’s Degree	Yes	Yes
W4	53	Female	Deputy Principal	34	Bachelor’s Degree	Yes	Yes
G1	30	Female	Head teacher special ed	4	Master’s Degree	Yes	No
G2	52	Male	Reliving deputy principal	17	Bachelor’s Degree	Yes	No
G3	55	Male	Principal	35	Master’s Degree	Yes	No
G4	46	Male	School counsellor	16	Postgrad Diplomas	Yes	No
O1	32	Female	School psychologist	5	Postgrad Diploma	Yes	No
O2	47	Female	Head teacher Special Ed teacher	11	Master’s Degree	Yes	Yes
P1	63	Female	Deputy Principal	40	Graduate Diploma	Yes	Yes
P2	38	Male	School counsellor	10	Graduate Diploma	Yes	Yes
P3	32	Male	Head teacher Special Ed	10	Master’s Degree	Yes	No
T1	58	Male	Principal	35	Master’s Degree	Yes	No
T2	43	Female	Deputy Principal	20	Graduate Diploma	Yes	Yes
T3	44	Female	Teacher	6	Master’s Degree	Yes	Yes
T4	27	Female	Teacher	3	Master’s Degree	Yes	No
T5	41	Male	Head teacher wellbeing	20	Bachelor’s Degree	Yes	No

The six schools included in the project were located in four towns in inner regional and outer regional areas of NSW. The town names were replaced with codes to ensure the participating schools would not be identified. Schools ranged in size from 300 to 1100 students. Many of the students’ families were low of low socio-economic status (SES) and had significant and complex needs, such as drug and alcohol dependence, homelessness, unemployment, and mental health issues, that limited their capacity to support their children’s health and educational needs. There was also a high proportion of student families from Indigenous backgrounds. The key demographic features of the schools reported in Table 2 are based on self-reports from the stakeholders interviewed in each School. Each of the schools was assigned a code to assure anonymity.

**Table 2:** School demographic features

Code	Total students	Aboriginal/Torres Strait Islander students	Students with disability or complex needs	Other major support needs
S1T1	750–780	225–235 (30%)	255–275 (35%)	None identified
S2T2	300	150 (50%)	60–70 (20–25%)	Mental health (45% of students)
S3T3	440	190 (43%)	180 (40%)	Very complex needs (5%)
S4T4	1000–1100	150–165 (15%)	100 (10%)	Increasing EAL/D students
S5T4	750–760	315–320 (42%)	200 (26%)	Additional 15 students with exceptionally complex needs
S6T4	700	210 (30%)	100 (14%)	Increasing EAL/D students

Finally, the communities in which participants lived were also characterised by financial disadvantage; the median household weekly incomes in each community were significantly lower than the NSW average. Table 3 contains demographic data selected from the 2016 ABS survey (Australian Bureau of Statistics, 2020), to highlight key characteristics of the local population that contribute to disadvantage. Data for the whole of NSW, the state in which each of the schools is located, have been provided as a point of comparison.

**Table 3:** Local area demographics

	Town 1 (T1)	Town 2 (T2)	Town 3 (T3)	Town 4 (T4)	NSW
ASGS remoteness classification	Inner regional	Outer regional	Outer regional	Inner regional	N/A
Population—general	34,339	4519	7984	33,885	
Population—5–19	6762 (19.7%)	849 (18.6%)	1426 (17.9%)	6604 (19.5%)	18.4%
Aboriginal/Torres Strait Islander	5420 (15.8%)	1179 (26.1%)	1198 (15.0%)	4190 (12.4%)	2.9%
Median age—general	35	44	39	38	38
Median age—Aboriginal/Torres Strait Islander	21	22	20	21	22
Median weekly household income—general	$1294	$807	$1167	$1121	$1486
Median weekly household income—Aboriginal/Torres Strait Islander	$1177	$858	$1036	$1008	$1486
One parent family	1865 (21.4%)	308 (28.8%)	397 (19.5%)	2001 (23.75)	310,906 (16%)

*Source* ABS 2016 Census QuickStats (Urban Centre Locality)

### Analysis

Thematic analysis, “a method for identifying, analysing, and reporting patterns (themes) within data” (Braun & Clarke, 2006, p. 79), was conducted in three stages. In the first stage, data samples were open coded by two members of the research team. Open coding is “identifying interesting features of the data in a systematic fashion, collating data relevant to each code” (Braun & Clarke, 2006, p. 87). Results from this initial coding were compared between the two, using Cohen’s Kappa, which is a metric that assesses the agreement between two raters (Bland, 2008). The comparison for these raters resulted in a high Cohen’s Kappa agreement (*κ* = 0.92; with 1.0 being the highest level of agreement possible). In the second stage, there was an exchange of the results of the coding over several meetings where the authors discussed the derived codes, sub-categories, and categories. Once agreement was achieved, all themes, categories, and sub-categories were carefully compared for any overlaps (Bengtsson, 2016). Triangulation of the data was achieved by involving all authors in every stage of the data analysis to ensure credibility, validity, and trustworthiness (Flick, 2018).

## Findings

The data analysis resulted in three key themes: (a) current approaches to wraparound and their strengths, (b) barriers to effective wraparound, and (c) additional resources needed for effective wraparound. Each of these themes is discussed in detail in the sections that follow.

## Current approaches to wraparound and their strengths

Although none of the schools had formalised wraparound teams, there were well-developed collaborative practices to support students with complex needs, described by Judy as, “Not so much a program… more a strategic approach”. However, a lack of resources means that support is often crisis driven. Another interviewee (Bella) described the approach to wraparound as, “We just work it out ourselves as we go along”.

In some cases, the schools operated as a hub for a range of external providers who could use school facilities to meet with students. In addition, a number of school staff are involved in liaising with external providers, helping students and their families to access services, and coordinating the provision of these services. School staff also collaborated with case workers from external organisations such as the NSW Department of Education, NSW Police, Juvenile Justice, Family and Community Services (FACS), and NSW Health, facilitating a coordinated approach to support. This sometimes occurred during organised meetings in which all the providers caring for students come together with school staff and student families to discuss progress. This key finding is well captured in the following from DCS3:

I guess I see school as the hub, bringing in stake holders. Often, we coordinate individual learning support team meetings. Either on a regular basis or during crisis periods in situational stress for families. Checking with kids from time to time. Support staff in creating support plans for students, whether they be learning needs, health care needs. Providing access or assisting families with accessing external support services. Following the legal obligations, getting the mandatory reporter guides.

### External collaborators

All of the Schools were extremely resourceful in identifying available sources of support and taking a leadership role in coordinating support from external collaborators. As Jonathan noted:

We generally do our utmost to fulfil the students’ needs both academically as well as socially, and considering lack of funds, I think, you know, we don’t ever turn anyone away…we generally work with all of the different agencies that we can utilise and it’s good, hard work from the people that actually come day to day…I think we do a pretty good job.

Multiple external agencies and providers were involved, including government agencies such as NSW Police, FACS, Juvenile Justice, Child and Adolescent Mental Health Services, and NSW Health. An extensive range of non-government organisations (e.g. Marathon Health, Mission Australia, Life without Barriers, and Uniting Care, amongst others) were also involved. Health providers, such as general practitioners (GPs), paediatricians, psychiatrists, psychologists, speech pathologists, and occupational therapists were also mentioned as supporting students with complex support needs at participating schools. Participants painted a picture of their schools accessing any and all available services, with the onus of knowing what services were available sitting largely with education professionals.

### School-based resources

In general, one of the great strengths in each of the schools was its committed and experienced staff. Many staff members juggled multiple roles as they sought to support their students. In addition, participants identified existing school-based resources as being integral to the provision of support for students with complex needs. Participants from five schools discussed the provision of targeted support for Indigenous students, including Aboriginal Education Officers/Advisers, who were supported by Clontarf for Boys and Girls Academy (not for profit organisations). Some schools also described having connections with local Aboriginal Elders or the Aboriginal Lands Council. T3, 4, and 5 explained:

The Opportunity Hub do a pretty good job…It’s with the Aboriginal Lands Council. They’re pretty good with – they call it the Opportunity Hub, so they have people that work with them. At the moment, they come in and do mentoring with Aboriginal boys, Aboriginal kids but we use them for the boys because we’ve got the Girls’ Academy, so they’ll send people out and do cultural awareness and look for, basically, opportunities to get the kids into.

School learning support units often acted as a hub for services, through case management, in addition to providing educational support, individual learning plans, emotional and social support, and referrals to external providers. Learning support unit staff included special education teachers and teacher’s aides. Special education staff also provided behaviour support, using a range of strategies, including behaviour management plans, time-out cards, and Check-In Check-Out interventions with senior staff. Some schools also received support from regional offices of the NSW Department of Education:

There is support through New South Wales public schools, so each school should have one, should have a learning and support coordinator, so the person kind of in charge of that. So, they cover access to curriculums, and the two arms of those are curriculum and welfare, which you’d understand, being a teacher. So, that person co-ordinates that, so I get referrals through that team as well at times, for either cognitive testing, if they’re performing poorly, or behavioural things if the parents are OK and they’re going to follow up with a paediatrician. That will be the co-ordination. (G4)

Other school-based resources mentioned by participants included school counsellors and/or psychologists, who worked directly with students with mental health needs, but also referred students to outside agencies. Interviewees also considered case managers to be important, but these duties were often shared between a range of staff, including members of the learning support unit, deputy principals, principals, and head teachers. DECS4 explained, “I need everybody feeding into the case management”. Some schools had a head teacher responsible for wellbeing or welfare.

Two of the schools had implemented restorative practice programs. A well-established restorative practice approach in one of the schools had brought immediate benefits: according to Henry, one of the school leaders who was interviewed, school suspensions were reduced by 50% in the first year after the program was introduced. Extensive training of staff in this school resulted in a high level of engagement in restorative practice, with extremely positive results across the board.

### Positive relationships with students and families

Another key aspect of the support provided to students with complex needs was the development of positive relationships with students and their families. One way that some of the participating schools reported fostering these relationships was by actively involving both the individual students and their family members in the development of individual plans. All stakeholders also described their schools as focussing on regular, constructive communication that is open, respectful, honest, and culturally sensitive, devoid of aggression and judgement, and including both positive and negative feedback. Staff in all of the schools discussed having made significant efforts to build rapport through home visits and phone calls. Finally, the introduction of restorative practice in one school had a very positive impact on breaking down generational mistrust of schools and building constructive relationships with students and families. Henry proudly described its effectiveness:

It has got those really hard parents, as well as the parents who were just letting things go, they have seen what we have got is a really fair process for dealing with all problems. We teach problem solving, formal circles, informal circles, where the perpetrators and the people who have been wronged, sit with each other, the parents there with them. We have everyday school kids 3 or 4 of those just randomly chosen. We have our school community, a couple of facilitators and a couple of support people if they need, a couple of Aboriginal support people. They all have their opportunity to have a say. It is a place where it is safe to have your say. We have never had a failure.

## Barriers to effective wraparound

Interviewees identified several barriers to effective wraparound support (e.g. lack of services, geography, support service staffing, student and family issues, communication, and school issues). The overarching barrier (mentioned by every participant) was a lack of access to medical and other support services provided by external collaborators/providers. One challenge in this regard was that even when the support was available, access could still prove to be difficult, as MaryAnn explained, because outside agencies often have very long waiting lists.

A lack of mental health services, particularly for youth, was evident throughout the interviews. Two interviewees (Michael, Laura) expressed concern that lack of access to specialist mental health services had led to GPs over-medicating because students could not access appropriate mental health care. Participants explained that although medical and other support services are found in inner regional areas, many parents of students at schools in outer regional areas could not afford the time or cost of travelling. As expressed by Henry, case workers were often located out of the area, negatively impacting their ability to provide timely responses in a crisis. Henry provided an example of a time when FACS was contacted because a student’s safety was in doubt, and the caseworker was located more than 400 km away. Overall, participants expressed that when external support services are available, they are often understaffed, caseworkers change frequently, and some staff are not adequately screened or trained for the roles they hold. Many external collaborators have unreliable funding sources and are not able to provide long-term solutions.

Another significant gap in supporting mental health of students with complex support needs was insufficient funding for school counsellors, resulting in school counsellors being “swamped” (as eloquently expressed by MaryAnn), and having to make difficult decisions about what to prioritise in their work. For example, Daniel elaborated:

I get a lot of referrals from teachers, parents, self-referrals from students, but it really comes down to, is there risk of significant harm? And there is risk of significant harm, and I’m often filling out the MRG [mandatory reporting guide], I’m taking kids over to the hospital who are suicidal, calling parents, obviously calling other services, trying to, you know, hook kids up and families up with other services, so there’s a fair social work type aspect to it at times.

Similarly, Bella stated: “Our counsellor is only a triage. She is here with us 3 days a week and that is all she has the capacity to do, is triage and assessment testing”.

There was also a perception amongst most interviewees (10) that funding models through the National Disability Insurance Scheme (NDIS) were problematic, as the provision of services was being informed primarily by whatever supports individual agencies had available, rather than focussing on meeting students’ needs. Participants T3, 4, and 5 lamented one student’s situation, “one child with cerebral palsy, the physiotherapist that she was seeing, that did go off NDIS, has stopped doing it, so now she doesn’t have anyone. It’s really tough”. Interviewees also cited problems with NDIS funding carrying across key transition points such as primary to high school, or high school to post-school. They also stated that communication with external collaborators (including government agencies) was often difficult or limited, causing problems with inter-agency coordination and information sharing. G1 discussed the fact that negative relationships between students and external agencies, particularly NSW Police and FACS, were a challenge to incorporating these agencies into wraparound support, “some students have experienced significant trauma with home life, of in and out of FACS care, involvement with police, and parents with drug and alcohol dependencies, and that type of stuff”.

MaryAnn cited inconsistent school attendance as a barrier to the provision of school-based care. Other participants (Jeanette, Michael, Laura) suggested that many students’ families were not equipped to support their children because they faced their own challenges, including lack of time or money, high stress levels, or complex needs of their own, such as drug or alcohol dependency.

Several interviewees revealed that some barriers seemed to be school-focussed. A lack of a dedicated school staff member/team for coordination meant that many staff in each of the schools were performing multiple roles. Other staffing issues mentioned were high staff turnover and difficulty in attracting teachers and other professional staff to work in inner and outer regional areas.

## Additional resources needed for effective wraparound

A wide range of needs were identified by the key stakeholders to support the effective implementation and provision of wraparound support, but school staffing, school facilities for holistic support, and streamlined funding models were mentioned most often.

### School staffing

All participating schools identified a need for more formalised coordination, preferably based at the school. Although a wraparound approach had been adopted by many of the schools, the consensus was that when a number of agencies were involved, services could become ad hoc because of the need for a dedicated person to take final responsibility for planning and coordination. As Judy noted, “I think it happens more incidentally than by design— or maybe we’re designing it as we do it, but it’s not institutional design or systemic design”.

### School facilities for holistic support

Participants at all schools expressed a desire to expand onsite facilities to provide space for external providers to see students at school. They had several different suggestions as to what kind of additional support could be offered at school: (a) after-school academic support; (b) drop-in/wellbeing spaces that included facilities (and funding) for practical support, for example showers, facilities to wash clothes, and meals; (c) parent education, (d) career advice, and (e) health care. MaryAnn suggested, “I would love to have some sort of wellbeing hub so we can have lots of experts here on a regular basis. You know, the facilities to be able to assist the kids with schoolwork”*.* Generally, interviewees wished for a greater focus on supporting families, for example by supporting them in managing their own disabilities, as well as issues such as drug and alcohol addiction or domestic violence. In addition, some parents required literacy support to better engage with the schools, health providers, and other agencies.

### Improved access to health care

Timely access to health professionals was considered essential by the interviewees to meet students’ health needs, but also for diagnosis so that funding for support can be obtained (Bella). In particular, schools identified the need for psychiatrists, psychologists, paediatricians, speech pathologists, and occupational therapists. Mental health, including support for misuse of drugs and alcohol was identified by many of the schools as an area of major need: “So, mental health I think would be a big number one, because there’s drug and alcohol…because of the location, and the epidemic of drugs in the community, so there’s big issues, what they bring from outside school into school” (Jonathan). Other participants (Kim, Wei, Sophie, James, Cherie) suggested a multi-disciplinary mental health hub be established on school premises to provide services such as general counselling and drug and alcohol counselling.

## Discussion

This study sought to determine the wraparound support needs of students with complex needs in regional schools, along with the enablers and barriers to effective wraparound support in regional schools. The support needs of students were mostly discussed within the theme of additional resources needed for effective wraparound, and participants primarily expressed that students and their families most required wraparound support in accessing medical and mental health services. This is very much in line with the exosystem of Bronfenbrenner’s Ecological Systems Theory (Bronfenbrenner, 1994), as when community-based family services were unavailable or difficult to access, school administration stepped in and supported the students and families, however, they could. When wraparound was successful, it was very much happening at the micro- and mesosystems levels, where students, families, and schools were working together to find support solutions (see Fig. 1).

This sense of collaboration and cooperation in the microsystem and mesosystem was present throughout the participating schools. School leaders, staff, and other stakeholders demonstrated incredible commitment and resourcefulness in providing what wraparound support they could to students with complex needs. One prime example of this was the implementation of restorative practice in one of the schools, an approach that was not only successful at the school level but had been adopted by the broader community as well. This is aligned with the findings of Augustine et al. (2018), who describe restorative practices as a way for a school to respond to conflict as a community as an alternative to exclusionary and punitive discipline practices. These alternative practices include:

…using affective statements to express personal feelings to build community, to formal practices, such as responding to a student’s disruptive actions in a ‘responsive circle’. In this circle, students and staff discuss the incident with the offender, being careful to emphasise the harm that was done rather than the person who did it. In this way, the offender is given time to reflect, apologise, make amends if necessary, and reintegrate into the community (Augustine et al., 2018, p. 1).

In many cases, the barriers or challenges the schools in this study faced were in line with the previous research. An analysis of literature conducted by Cumming et al. (2019) identified the following four factors as the most common barriers to effective wraparound programs: (1) Lack of collaborative functioning of the wraparound team, with differences in culture, priorities, and protocols creating difficulties with communication and information sharing; (2) insufficient funding and resource allocation, (3) a lack of strong, effective and open-minded leadership of principals, facilitators, and key decision makers, (4) aspects of the wraparound program, such as the stage at which students were recruited to participated, the cultural appropriateness and adaptability of the program, the length of the program, the intensity of the wraparound services, consistency of personnel, student and family engagement with the program, and the care offered after exit, were noted as important to overall wraparound efficacy.

Of these factors, several were also identified by the schools as being major barriers, including a lack of (a) collaborative functioning, (b) formal understanding between team members, (c) planning and groundwork, (d) communication, (e) a dedicated coordinator/coordination team, (f) funding, and (g) issues relating to student absenteeism or staffing changes in the wraparound team. Some participants also noted that the inconsistency of personnel, unavailability of services, and/or lack family engagement constrained the provision of effective wraparound.

Many of the challenges faced by the Schools stem from systemic factors in the macrosystem and exosystem that cannot be addressed at the local level, whereas others may be overcome with sufficient funding and resources at the individual School level. For example, the difficulty of accessing medical services such as paediatricians, psychologists, mental health services, and other allied health professionals such as speech pathology, hearing and vision services, and occupational therapy for students with complex needs, reflects a general lack of availability of these services in the wider community. Significant funding increases are required to ensure adequate and equitable access to medical services in many rural or remote communities.

Similarly, the frustration expressed by key stakeholders about what they perceived to be unnecessarily complex and unreliable funding models are the result of well acknowledged issues in the funding and delivery of services for disability that have impact beyond schools. Finally, the need for greater funding at the school level to employ sufficient staff to meet students’ complex needs, particularly for coordination of wraparound, cannot be addressed easily by individual school leaders.

## Recommendations

There are strategies that can be adopted at both the systemic level and the school level that would allow schools to increase the effectiveness of their current approach to wraparound using their existing resources. The following section provides recommendations targeted at both levels, based on the results of the current study and best practices noted in the literature.

### Systemic

#### Funding

There is a desperate need for a more coherent and coordinated approach to wraparound. In the current study, wraparound coordination was shared amongst several staff members in each of the participating schools, with external providers also contributing. The claim from participants that funding for a dedicated school-based case manager would greatly enhance the effectiveness of wraparound services is strongly supported by a review of literature conducted by Cumming et al. (2022), who identified sufficient funding as a major enabler of effective wraparound programs. Teams should be sufficiently funded to allow the investigation of all possible avenues of support and the development of strong working relationships with external collaborators. Having one person or a small team consistently performing the coordination role would also likely lead to better collaboration with students who have suffered trauma or have attachment difficulties (Hill, 2018).

#### Staffing

Attracting and retaining staff posed a major challenge for the schools in this study, in line with reported high staff turnover rates in rural and remote schools (Halsey, 2018). The experiences of the participating schools suggest that current NSW Department of Education policy and incentives for attracting and retaining staff to rural and remote schools needs to be rethought. It may be helpful to collect detailed data about staffing levels, rates of retention, time taken to recruit staff, etc., to present a compelling case to the NSW Department of Education for incentives to be extended.

#### Improved access to health care

The lack of access to health care in rural and remote Australia is a well-known problem and has significant implications for health outcomes. The inability for students to access appropriate health care reported by each school reflects a lack of access to health care in the general community in rural and remote communities. For example, the Australian Institute of Health and Welfare reported that, whilst the numbers of general practitioners per capita in rural and remote locations were higher than in metropolitan areas, there were far fewer medical specialists and other medical professionals such as psychologists, physiotherapists, occupational therapists, optometrists, and dentists per capita, with the reduction in numbers greater as the degree of remoteness increased (Australian Institute of Health & Welfare, 2019).

However, given the close connection between health and educational outcomes, there is an argument that improved access to health care would also help to reduce inequities in educational outcomes (Australian Institute of Health & Welfare, 2019). This study has highlighted the high incidence of complex needs in students at six schools, including disabilities and behavioural and mental health issues. Yet access to adequate health care for these students is woefully inadequate. Without sufficient support for students’ health needs, wraparound programs, regardless of how well they are coordinated at the school level, cannot be effective. The NSW Government’s figures show that the current ratio is one counsellor for every 743 students across the state, which is, according to the president of the NSW Teachers Federation, deeply concerning (NSWTF, 2020). The Federation conducted its own survey with 5300 teachers and principals. Only 20% of participants stated that their school had a school counsellor on site daily. This was consistent with the present study in that many interviewees mentioned that they had a school counsellor on site only 4 days a week. Given the Schools’ sizes, this is a critical problem that needs to be addressed.

Whilst this problem extends well beyond the scope of the education system, it is systemic and needs to be addressed. Greater funding for health professionals in rural and remote areas is the first step, however, another avenue that may support access is the provision of onsite health facilities in schools (Caldas et al., 2019). This would likely alleviate the communication difficulties that many schools reported when dealing with external providers such as health professionals. It would have the further advantage of removing barriers related to travel for students and their families.

Another strategy would be to expand the provision of telehealth services. As a result of COVID-19, there have been significant advances in the provision of telehealth in a range of services. This has been accompanied by a growing research interest in the delivery of health services and evidence of positive outcomes. For example, Langbecker et al. (2019) found that the majority of primary school children in five rural and remote areas that received occupational therapy and speech therapy via telehealth, showed improvements in speech and language skills, educational outcomes, and class participation after one semester.

### School-Level strategies

#### Professional development

Several areas where professional development would enhance the capacity of all school staff to support students with complex needs were identified by stakeholders. Providing all school staff, irrespective of their role, with training in trauma-informed practice, restorative practice, and disability support would ensure a consistent whole-school approach. Together with resilience training, this may also support staff wellbeing and reduce staff turnover, which were two areas of concern raised in interviews. Although funding would improve the capacity of the schools to provide professional development, there is also scope for the existing expertise with schools to be shared in a Community of Practice approach. It may be possible for several schools within regions to collaborate on providing in-house staff training and development workshops. There may also be capacity for additional training and development to be accessed through the Department of Education through a distance learning approach.

#### Use of technology to support collaboration

A key aspect of wraparound is planning and coordination between different stakeholders, including education, health, relevant government departments, and students and their families. In rural and remote locations, it may be difficult to coordinate physical meetings, particularly with visiting health professionals or representatives of agencies located in other areas. However, coordination is still possible using online video-conferencing systems, which have become more common as a result of COVID-19. Utilising technology in this way would allow for more regular meetings of stakeholders without the inconvenience of travelling large physical distances. The success of this approach will rely on the implementation of case management officers/teams within the Schools, who have the responsibility and capacity for coordination.

## Conclusion

This study explored the perceived wraparound support requirements of students with complex support needs attending schools in rural and remote New South Wales. The findings indicated several strengths of the current bespoke approaches to wraparound in rural and remote schools. Furthermore, several barriers and needs were also recognised. The recommendations provided by the authors (see above) have a great potential to enhance the capacity of rural and remote NSW schools to provide wraparound support for students with complex support needs.

There are currently few studies that explore wraparound supports in rural and remote New South Wales. Our findings therefore contribute to the literature by providing additional insights into wraparound supports in these locations that may inform future practices. As a qualitative approach was employed where diverse stakeholders were interviewed, the data collected allowed for rich description and a more contextualised understanding of existing wraparound supports. More research is needed in this area, including that which captures the voices of the students and their families, which was not possible in this study. The findings from this research, however, enabled insights into several possible strategies that could enhance the capacity of rural and regional NSW schools to provide wraparound support for students with complex needs.
